# Molecular evolutionary rates are not correlated with temperature and latitude in Squamata: an exception to the metabolic theory of ecology?

**DOI:** 10.1186/s12862-016-0666-4

**Published:** 2016-05-20

**Authors:** Jonathan Rolland, Oriane Loiseau, Jonathan Romiguier, Nicolas Salamin

**Affiliations:** Department of Ecology and Evolution, Biophore, University of Lausanne, 1015 Lausanne, Switzerland; Swiss Institute of Bioinformatics, Quartier Sorge, 1015 Lausanne, Switzerland

**Keywords:** Diversity, Reptiles, Mutation rate, Snakes, Lizards, Speciation rate

## Abstract

**Background:**

The metabolic theory of ecology stipulates that molecular evolutionary rates should correlate with temperature and latitude in ectothermic organisms. Previous studies have shown that most groups of vertebrates, such as amphibians, turtles and even endothermic mammals, have higher molecular evolutionary rates in regions where temperature is high. However, the association between molecular evolutionary rates and temperature or latitude has never been tested in Squamata.

**Results:**

We used a large dataset including the spatial distributions and environmental variables for 1,651 species of Squamata and compared the contrast of the rates of molecular evolution with the contrast of temperature and latitude between sister species. Using major axis regressions and a new algorithm to choose independent sister species pairs, we found that temperature and absolute latitude were not associated with molecular evolutionary rates.

**Conclusions:**

This absence of association in such a diverse ectothermic group questions the mechanisms explaining current pattern of species diversity in Squamata and challenges the presupposed universality of the metabolic theory of ecology.

**Electronic supplementary material:**

The online version of this article (doi:10.1186/s12862-016-0666-4) contains supplementary material, which is available to authorized users.

## Background

Defining what mechanisms explain the current distribution of species on Earth is an important question in evolutionary biology and ecology. Although this question has not yet been settled [1, 2], hypotheses proposing temperature as the main driver of global patterns of species diversity have received considerable attention [3, 4].

Temperature is a key ecological factor that is commonly used to describe the spatial distribution of species, but it is also supposed to have a profound influence on the metabolic rate, and thus the mutation rate, of ectothermic species [1, 5]. This has lead to the formulation of the “metabolic theory of ecology” [5–7] that predicts accelerated rates of molecular evolution (substitution rates), increased genetic divergence between populations, faster speciation rates and ultimately higher species richness when temperature is higher [8]. This theory is closely related to the hypothesis of evolutionary speed, which describes the increase in temperature in the context of the latitudinal diversity gradient [9]. Mechanistically, higher metabolic rate should increase the rate of cell division leading to the fixation of mutations during the DNA replication process and should also produce more oxygen free-radicals that directly damage DNA [10, 11].

A positive relationship between the rate of molecular evolution and temperature (or latitude) has been shown in a large number of ectothermic organisms such as plants [12–14], foraminifera [6], fishes [15], amphibians [16] and turtles [17]. Unexpectedly, this positive relationship was also found in endotherms, such as hummingbirds [18] and mammals [19, 20], but has since been invalidated for birds as a whole [21]. Although there are no *a priori* reason for metabolic rates to be related with environmental temperature in endotherms because of their constant body temperature, Brown *et al*. [5] proposed that indirect factors could also favor endotherms diversification where temperature is higher, such as Red Queen effects, through interactions with ectotherms, and ecosystem productivity.

The prediction of the metabolic theory of ecology that molecular evolutionary rates should be positively correlated with temperature has been validated on all the clades of ectothermic organisms that have been studied so far. This has however never been tested on Squamata, which is one of the main group of vertebrates. Here, we tested the link between the rate of molecular evolution, temperature and latitude in Squamata and found that it may be an exception to the rule.

## Methods

### Phylogeny

We used the most recent non-dated phylogeny available for all Squamata [22] based on 9 nuclear and 3 mitochondrial genes. In this study, 12 DNA regions were concatenated into a supermatrix and the molecular phylogeny was built with maximum likelihood (RAxMLv7.2.8 [23]) using the GTRCAT approximation of the GTR + Γ substitution model. This phylogeny contains 4,161 species out of the more than 9,400 extant species of lizards and snakes (~45 %, *sensu* [22]).

### Species distribution maps and climatic data

We downloaded the distribution maps of 1,651 species (~17.6 % of all squamata) found in the phylogeny from the IUCN website (http://www.iucnredlist.org). We plotted species distributions and estimated the mean latitude for each species with the R package *raster.* We also calculated the mean annual temperature for each species from climate spatial grids downloaded from the WorldClim website (http://www.worldclim.org [24]) at a resolution of 2.5 arc minutes.

### Sister species comparisons of molecular evolutionary rates, temperature and latitude

In the non-dated phylogenetic tree of Pyron *et al.* [22], branch lengths were expressed as the number of substitutions per site. We decided to compare the contrast of rates of molecular evolution with the contrast of temperature and latitude between phylogenetically independent pairs of species. Following recent studies [19, 20], we chose to compare only species living at different temperatures, with a minimum difference of 5.3 °C between the two species (this threshold corresponded to the squared deviation of the temperature of all species). We used the branch lengths (from each species to their common ancestor, following [19]) as an estimator of the molecular evolutionary rates. As the times between each of the two sister species and their common ancestor are the same, we did not need to calibrate the tree to compare the molecular evolutionary rates between species. We tested whether the rates of molecular evolution were accelerated at higher temperature for each pair of species. To do so, we could not use Phylogenetic Generalized Least Squares (PGLS) model because the data represents, on one side, branch specific measures (i.e. molecular rates) and on the other side, species traits at the tips (i.e. temperature and latitude). Instead, to select phylogenetically independent pairs of sister species in the tree, we decided to implement a new algorithm similar to the approach of Maddison [25] (code available upon request). Our algorithm contained four steps: 1. We assigned the mean temperature and the absolute mean absolute latitude to the species at the tips of the phylogenetic tree. 2. We sampled randomly one species and one of its closest relatives at the tips of the tree (these had a minimum temperature difference of 5.3 °C). 3. We recorded the two temperature values of the species, the two latitude values, and the two branch lengths from the most recent common ancestor to the species tips. 4. We then repeated steps 2 and 3. At each repetition, we made sure that the new species pair was not included in any of the clade previously sampled. When all the independent clades of the tree were sampled, we stopped the algorithm. Because of the random sampling at step 2, some species comparisons were not studied during this procedure. We thus repeated the algorithm to sample different sets of species pairs and assess if this could affect the trends obtained. To ensure good statistical power, we kept 1,000 algorithm runs with temperature, latitude and branch lengths for at least 50 species pairs.

For each of these replicates, and following the approach of Weir and Schluter [20], we tested with major axis regressions (*slma* function in *smart* R package) whether warmer branches were longer than cooler branches. If there is a positive relationship between temperature and branch length, the slope of the regression of warmer on cooler branch lengths should be greater than 1. With the same species pairs, we then assessed whether the difference of absolute latitude separating species was associated to rates of molecular evolution. To do this, we used a standardization of the branch length presented in [20]: we measured the difference between the branch lengths of the two species divided by the average of the two branch lengths. We used major axis regressions between these standardized branch length and the difference in absolute latitude between the two species. If there is an effect of the latitude on molecular evolutionary rates the slope should be different than 0. For all major axis regressions, we then plotted the slopes corresponding to these 1,000 relationships and estimated the number of significant relationships according to the number of relationships for which the lower boundary of the confidence interval was greater than 1.

## Results

### Diversity gradients of squamates

**Fig. 1:**
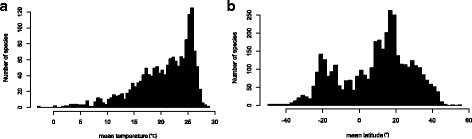
Number of species as a function of **a** temperature or **b** mean latitude in Squamata

Diversity was positively associated with temperature (Fig. 1a). However, unexpectedly, we found that diversity of squamates peaked in mid-latitude regions (Fig. 1b), with more species at 20° of latitude (~200 spp.) than near the Equator (~75 spp. at 0°).

### Sister species comparisons of molecular evolutionary rates, temperature and latitude

**Fig. 2:**
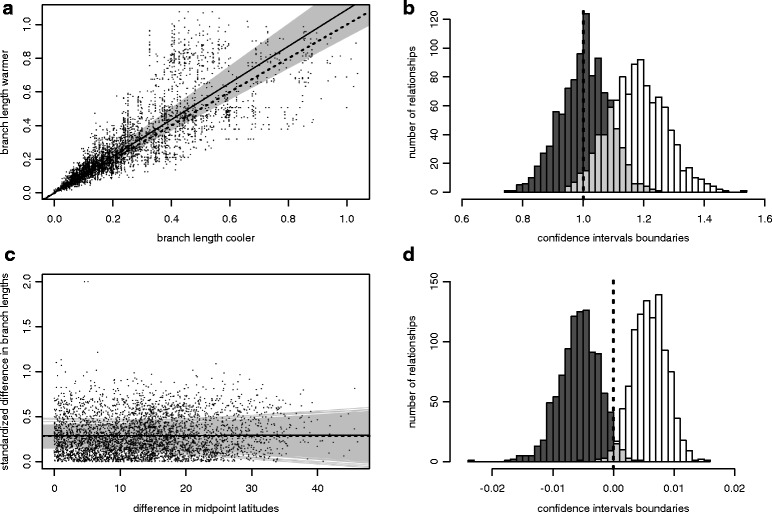
Relationships between branch lengths, temperature and latitude. **a** Major axis regressions drawn between the branch length of the warmer and the cooler species of the species pair. **b** Distribution of the lower (*black*) and the upper (*white*) boundaries of the confidence interval for the 1,000 major axis regressions presented in (**a**). **c** Major axis regressions drawn between the standardized difference of branch lengths and the difference of absolute latitude in the species pair. **d** Distribution of the lower (*black*) and the upper (*white*) boundaries of the confidence interval for the 1,000 major axis regressions presented in (**c**). In **a** and **c** grey lines represent the 95 % of the slopes of 1,000 relationships. Solid lines represent the median slope of the 1,000 relationships and dashed lines were the reference slopes (slope = 1 for (**a**) and (**b**), and slope = 0 for (**c**) and (**d**)), respectively

Among our 1,000 replicates, the mean slope of the major axis regression between warmer and cooler branches was 1.09 (median slope = 1.09, 95 % CI: 0.92 to 1.25, drawn from 51 to 141 species pairs, Fig. 2), indicating no significant effect of the temperature on branch length. The lower boundaries of the confidence interval of the slope estimate were higher than 1 in 544 of 1,000 relationships (and the lower boundary was lower than 1 in 24 relationships). We also found no association between differences in absolute latitude and standardized branch length (median slope = 1.79 × 10^-4^, 95 % CI: 5.59 × 10^-3^ to 5.35 × 10^-3^; y-intercept = 0.287). Only 15 of the 1,000 relationships had a confidence interval of the slope estimate with a lower boundary higher than 0 and 30 had upper boundaries of the confidence interval lower than 0. We found similar results when we compared the branch lengths of the species distributed at higher latitude with those of the species at lower latitude (Additional file 1: Figure S1). Similar results were also found when we considered a difference of at least 10 °C between the two species of the sister species pair.

## Discussion

Several recent studies have shown that molecular evolutionary rates are correlated with mean temperature in many organisms, such as in mammals [19], amphibians [16], and flowering plants [12, 14], providing empirical support for the metabolic theory [5]. These previous studies were in line with the hypothesis that molecular evolutionary rates, through their potential correlation with speciation rates [9], were responsible for the construction of the latitudinal diversity gradient. In contrast, we show here that, in Squamata, higher molecular evolutionary rates are not associated with higher temperature or lower absolute latitude.

Previously, the few studies that have shown that molecular evolutionary rates and temperature - or latitude - were not associated, were focusing on endothermic taxa (such as birds [21]). To our knowledge, our study is the first to demonstrate an absence of relationship in ectothermic taxa. According to the predictions of the metabolic theory of ecology there should be a positive relationship between molecular evolutionary rate and temperature (or latitude) in ectothermic species, because their body temperature depends on external temperature [6]. Given the size of the data used here, it is very unlikely that we lack statistical power to detect any significant relationship. It is also unlikely that our conclusions could be affected by methodological artifacts, such as the under-estimation of multiple substitutions in long branches. This artifact may appear when the two members of the species pair belong to clades with large differences in species richness. Because short branches, which are more likely in species-rich clades, are less affected by saturation due to multiple substitutions, the sum of their branch lengths will more accurately represent the true amount of molecular evolution [26]. In contrast, species-poor clades will on average have longer branches, which could be underestimated by phylogenetic reconstruction methods and thus lower our estimates of evolutionary rates. As richness in squamata peaked at high temperature (Fig. 1a), the presence of this node density effect would lead to substantially higher evolutionary rates at high temperatures. As we observe no significant relationship between temperature and evolutionary rates, this possible bias is conservative. Overall, we have greater confidence to affirm that our results cast doubt on the universality of the metabolic theory of ecology and the evolutionary speed hypothesis, as a major explanation to the patterns of species diversity in vertebrates.

There are several other potential explanations for an absence of relationship between molecular evolutionary rates and temperature (or latitude). First, the molecular evolutionary rates of squamates may be particularly high in mid-latitude regions, where richness is higher (Fig. 1). Indeed, high speciation rates – potentially related to increased molecular evolutionary rates, but not necessarily – may be found in low-rainfall regions, e.g. deserts, such as at the boarder of the Hadley cell – between 20 and 30° of latitude. However there are two reasons why we think that this hypothesis is unlikely. First, we did not find any study supporting this hypothesis in the literature and, second, applying a quadratic effect on absolute latitude did not improve the relationship between absolute latitude and molecular evolutionary rates (results not shown).

A second hypothesis for an absence of relationship between molecular evolutionary rates and temperature (or latitude) is that Squamata have a particular biogeographical history that masks the relationship between molecular evolutionary rates and temperature. If dispersal rates have been high from the temperate regions to the tropics, species currently found in tropical areas may have recently colonized the tropics and may have experienced an increase of molecular evolutionary rates only recently. As we measured a mean molecular evolutionary rate from the most recent common ancestor of the sister pairs to the tips of the phylogenetic tree, we might not detect this recent increase of molecular evolutionary rates in tropical lineages (this bias has already explained in [19]). This hypothesis is consistent with the recent findings of Pyron [27], suggesting that the diversification of Squamata have been high in temperate regions and that expansions of lineages from the temperate regions toward the tropics have been frequent. These events of dispersal may thus have blurred the relationship between evolutionary rates and temperature (or latitude). This also suggest that present day distributions alone might not be appropriate for testing the validity of the metabolic theory of ecology. This argument is nonetheless unlikely because it must concern a very wide number of lineages.

A third hypothesis is that life history traits (body size or generation time) may be the main drivers of substitution rates of squamata. Indeed, species with short generation times are expected to feature a higher number of replication cycles and mutations per unit times. Such relationships between life history traits and rates of molecular evolution were found in reptiles [28] and could prevent detection of a temperature/latitude effect.

To our knowledge, our study is the first to show that global diversity of squamata is peaking at mid-latitude. However, it is very difficult to know if this geographical pattern of richness is biased with latitude. It is possible that the number of species in humid tropical areas is underestimated, and this may slightly bias the distribution of species at low latitude. However, the literature provides evidence that squamata have very different distributions than birds and mammals with peaks of diversity in arid regions rather than in tropical wet forest [29, 30]. We have also no reason to think that desert regions (where we detect a high diversity of squamata) were much more sampled than tropical humid areas because both regions have low human population density (and likely low sampling probability). We thus have confidence on our results, and we suggest that further studies will refine the description of the diversity pattern of squamata.

## Conclusions

In this study, we show that the molecular evolutionary rates of Squamata are not associated with temperature and latitude. We propose that these results might be due to a particular evolutionary history of the group, leading to the formation of a reversed latitudinal diversity gradient with higher diversity at mid-latitude. Another explanation for the absence of relationship might be that the metabolic theory of ecology and the evolutionary speed hypothesis is not as pervasive as first believed. Future studies will have to determine the causes and the particularities of the latitudinal gradient of squamate species diversity.

## Additional file

Additional file 1: Figure S1. Relationships between branch lengths and absolute latitude. (a) Major axis regressions drawn between the branch length of the species at higher and the branch length of the species at lower latitude (for each species pair). (b) Distribution of the lower (black) and the upper (white) boundaries of the confidence interval for the 1,000 major axis regressions presented in (a). Among our 1,000 replicates, the mean slope of the major axis regression between branches at higher and at lower absolute latitude was 1.1 (median slope = 1.09, 95 % CI: 0.77 to 1.49, drawn from 51 to 141 species pairs), indicating no significant effect of the absolute latitude on branch length. The lower boundaries of the confidence interval of the slope estimate were higher than 1 in 696 of 1,000 relationships (and the lower boundary was lower than 1 in 304 relationships). (DOC 1126 kb)
