# Supplementary material for: Molecular evolutionary rates are not correlated with temperature and latitude in Squamata: an exception to the metabolic theory of ecology?
Source: BMC Evol Biol. 2016 May 20;16:95. doi: 10.1186/s12862-016-0666-4 (PMC4874019; doi:10.1186/s12862-016-0666-4)
Supplement: Additional file 1: Figure S1. — Relationships between branch lengths and absolute latitude. (a) Major axis regressions drawn between the branch length of the species at higher and the branch length of the species at lower latitude (for each species pair). (b) Distribution of the lower (black) and the upper (white) boundaries of the confidence interval for the 1,000 major axis regressions presented in (a). Among our 1,000 replicates, the mean slope of the major axis regression between branches at higher and at lower absolute latitude was 1.1 (median slope = 1.09, 95 % CI: 0.77 to 1.49, drawn from 51 to 141 species pairs), indicating no significant effect of the absolute latitude on branch length. The lower boundaries of the confidence interval of the slope estimate were higher than 1 in 696 of 1,000 relationships (and the lower boundary was lower than 1 in 304 relationships). (DOC 1126 kb) [file 12862_2016_666_MOESM1_ESM.doc]

**Molecular evolutionary rates are not correlated with temperature and latitude in Squamata: an exception to the metabolic theory of ecology?**

***-***

Supplementary Material

**Figure S1. Relationships between branch lengths and absolute latitude.** (a) Major axis regressions drawn between the branch length of the species at higher and the branch length of the species at lower latitude (for each species pair). (b) Distribution of the lower (black) and the upper (white) boundaries of the confidence interval for the 1,000 major axis regressions presented in (a). Among our 1,000 replicates, the mean slope of the major axis regression between branches at higher and at lower absolute latitude was 1.1 (median slope = 1.09, 95% CI: 0.77 to 1.49, drawn from 51 to 141 species pairs), indicating no significant effect of the absolute latitude on branch length. The lower boundaries of the confidence interval of the slope estimate were higher than 1 in 696 of 1,000 relationships (and the lower boundary was lower than 1 in 304 relationships).
